# New and Original Theories of the Great Physical Forces

**Published:** 1879-03

**Authors:** 


New and Original Theories of the Great Physical Forces. By Henry Raymond Rogers, M.D. Published by the Author.

This contribution to solar and terrestrial physics is a philosophic dissertation upon the influences and relations of "the forces" to the various physical phenomena and their causation of disease. Some of the premises are rather unique, probably, but still a perusal of the work will prove interesting.

